# Evolutionary correlation of water-related traits between different structures of *Dendrobium* plants

**DOI:** 10.1186/s40529-020-00292-4

**Published:** 2020-05-16

**Authors:** Mei Sun, Chun-Hui Feng, Zhen-Ya Liu, Kun Tian

**Affiliations:** 1 National Plateau Wetlands Research Center, Southwest Forestry University, Kunming, 650224 Yunnan China; 2 Ecological Research Station of Dianchi in Yunnan, Jinning, 650600 Yunnan China

**Keywords:** Pseudobulbs, Water-related traits, Drought endurance strategies, Coordinated adaptation, Water stress

## Abstract

**Background:**

Leaf water conservation and pseudobulb water storage are two of the strategies commonly employed by epiphytic plants to adapt to dry environments. During the flowering period, a great deal of water transpires through the flowers, which then influences water-related processes. However, there is little research on the coordinated relationship between the different structures of epiphytes. Our study explored the phylogenetic conservation and evolutionary correlations between structural traits of 8 species in the genus *Dendrobium* by using phylogenetic independent contrast (PIC) analysis.

**Results:**

Leaf dry mass, leaf water content, leaf dry matter content, specific leaf area, stomatal density, stomatal area index, pseudobulb length, pseudobulb width, and flower dry mass show strong phylogenetic signals. Pseudobulb length is significantly positively correlated with stomatal volume but significantly negatively correlated with mesophyll thickness according to both species mean values and PIC values. Pseudobulb internode length is also positively correlated with stomatal volume but negatively correlated with stomatal density according to PIC values. Pseudobulb width is significantly positively correlated with leaf dry mass, stomatal density, stomatal area index, flower petal vein number and flower dry mass but negatively correlated with specific leaf area according to species mean values. However, these correlations are insignificant when PIC values are analyzed. Stomatal volume is positively correlated with flower dry mass, and after phylogeny is considered, this correlation is still significant. Leaf dry mass is positively correlated with flower petal vein number according to species values. Flower number per pseudobulb is negatively correlated with upper epidermal cell size according to species values but negatively correlated with stomatal area index according to PIC values. There are no correlations between pseudobulb and flower water-related traits according to PIC values.

**Conclusions:**

A trade-off should exist in epiphytic plants between the two drought-tolerant strategies of pseudobulb storage and leaf water retention. Plants possessing thick blades with a few large stomata tend to use the pseudobulb water storage strategy to adapt to drought. Small flowers and low flower dry mass should be associated with the leaf water retention strategy. In addition, flowers and leaves exhibit an obvious water balance and should share common selection pressures. The present study provides a case with which to understand the coordinated adaptation of different structures in epiphytic plants.

## Background

Epiphytic plants mainly grow on rocks or tree trunks next to creeks and valleys, and many of them are crassulacean acid metabolism (CAM) plants or concomitant C3 and CAM plants (Silvera et al. 2009; Zhang et al. 2014). In contrast to those affecting regular terrestrial plants, water and nutrient limitations are the main factors that affect the survival, growth, and distribution of epiphytic plants (Zotz and Bader 2009a), and a lack of water is postulated to be the greatest stressor (Laube and Zotz 2003; Bartels and Chen 2012). However, epiphytes often show strong drought tolerance and can effectively adapt to a water-deficient environment (Watkins and Cardelús 2012; Sanger and Kirkpatrick 2017; Tay et al. 2019). Some employ CAM photosynthetic pathways to reduce water loss through nocturnal uptake of CO_2_ (Silvera et al. 2009; Zhang et al. 2014). Most epiphytes have specialized morphologies and structures that promote water retention, such as succulent leaves, rhizomes or specialized water storage tissue (Reyes-García et al. 2008; Yang et al. 2016). Studying the ecological adaptation strategies of epiphytes to water deficit is important for understanding the material flow, energy flow, and development of tropical and subtropical forest ecosystems (Jiang et al. 2014; Sanger and Kirkpatrick 2017).

Epiphytes usually have succulent leaves and relatively well-developed pseudobulbs. Coordinated functional traits, including leaf water conservation (Sun et al. 2014) and water retention in pseudobulb “reservoirs” (Yang et al. 2016), are two common drought tolerance strategies in epiphytes. A thicker epidermis and denser epidermal cells can effectively prevent water evaporation after the stomata are closed (Riederer and Schreiber 2001); the thick succulent mesophyll can also store more water than regular mesophyll (Zotz and Bader 2009b). Epiphytes also have a strong ability to absorb water from fog and dew, which is due to their thinner layer of vascular tissue and the thickness of the cuticle (Gotsch et al. 2015). The correlation of these structural traits effectively promotes epiphyte adaptation to drought. At the same time, during their evolutionary process, there should be a trade-off between water conservation strategies. Because leaves are the main structure used for photosynthesis, water loss by photosynthetic transpiration is still the main pathway for water loss in plants. Therefore, under extreme drought conditions, many epiphytes first reduce the surface area available for transpiration by shedding leaves. The pseudobulb is another important structure in drought resistance. Under a set of conditions that lead to leaf loss, water loss from the pseudobulb is only 30% of that lost under conditions where the leaves remain intact (Zotz and Tyree 1996; Li and Zhang 2019). Water, organic matter, and inorganic salt storage in the epiphytic pseudobulb has been shown in several previous publications (Ng and Hew 2000; Yang et al. 2016). The larger the pseudobulb is, the thicker the tissues and the greater the storage capacity. However, only a few studies have considered whether there is a correlation between leaf water retention and pseudobulb water retention. Studying how epiphytes maintain the balance between leaf water retention and pseudobulb water retention would allow us to learn more about the survival strategies of epiphytic plants.

Flowers are the reproductive structures of plants and determine the ability of plants to survive and spread. Changes in flower traits are closely related to pollination methods and abilities (Weber and Goodwillie 2013), and these traits show adaptations to changes in the external environment (Zhang et al. 2017). Although flowers contribute little to photosynthesis and are present for only a short period of time, a great deal of energy is expended during the flowering process, and a large amount of water is lost through transpiration (Lambrecht et al. 2011; Roddy and Dawson 2012; Teixido and Valladares 2014). However, if the water supply is insufficient during the flowering process, flowers will easily wilt, affecting the spread of pollen. Therefore, plants must maintain the water balance and osmotic pressure within the flowers during this period (Zhang et al. 2017). In addition, there is coordination between flower water-related traits, similar to the widely published conclusions about leaves. For example, similar to the positive correlation between leaf longevity and leaf weight, there is a significant positive correlation between flower longevity and flower weight (Zhang et al. 2017). However, previous studies have found that during the evolutionary process of plants, the water-related traits of flowers and leaves evolved independently of one another and that there are no correlations between them (Roddy et al. 2013; Zhang et al. 2017). Different types of plants show different trait relationships due to variation in their growth environment and other factors. At present, there are relatively few studies on the evolutionary correlations between different plant structures, which still need to be further explored in different plant groups (Roddy et al. 2016). To understand the long-term survival strategies of plants, it is of great significance to understand the relationship between different structures of plants and to understand the coordination between different plant structures as part of the whole plant.

*Dendrobium* is a typical epiphytic genus containing approximately 1000 species worldwide, including 78 species in China, mainly distributed in tropical and subtropical forests, with a higher distribution in karst forests. Plants in this genus are entirely epiphytic and show strong drought tolerance. Most members of the genus have fleshy leaves and stems, relatively thick cuticles, and a high water content throughout the plant (Sun et al. 2014). Flowers of this genus are bright, colorful, and fragrant, with a relatively fleshy texture and a long flowering period. Previous studies on the relationships of water-related traits between different plant structures in this genus found that the leaf and flower traits of *Dendrobium* are commonly affected by the environment and phylogeny (Sun et al. 2014, 2019). Evolutionary relationships between traits and coordinated regulation of plant functions are employed to cope with water deficits (Sun et al. 2014, 2019). *Dendrobium* use two strategies for water retention: leaf water retention and pseudobulb water “reservoirs” to maintain an equilibrium in plant water distribution (Yang et al. 2016). There is a significant positive correlation between flower longevity and flower weight in *Paphiopedalum*, but there are no evolutionary correlations between similar leaf traits (Zhang et al. 2017). In this study, eight species of *Dendrobium* were selected. Through an investigation of the morphology, structure, size, water content, and dry matter content of pseudobulbs, leaves and flowers and through the construction of a phylogenetic tree, we used phylogenetic independent contrast (PIC) analysis to explore the evolutionary relationships between different structures of *Dendrobium* and the biological strategy of coordinated evolution of different structures to adapt to water-deficient environments. A hypothesis was proposed that phylogeny should influence water-related trait variation and that water-related stress should induce some trait relationships between the pseudobulbs, leaves and flowers of *Dendrobium* species. This study will further verify the factors that affect the different structures of epiphytic plants and aims to further understand the coordinated evolutionary relationship between different structures of epiphytic plants.

## Materials and methods

### Study location and plant materials

The study was carried out in the wild orchid garden of the Xishuangbanna Tropical Botanical Garden of the Chinese Academy of Sciences (XTBG; latitude: 21.41°, east longitude: 101.25°; elevation: 570 m). XTBG is geographically located on the northern edge of the tropical region, on the gourd-shaped island formed by the Luosuo River, a tributary of the Lancang River, around Menglun town, Xishuangbanna. The area is atmospherically influenced by both Indian Ocean monsoons and East Asian monsoons. The dry and wet seasons are distinct, and the year is divided into a typical rainy season (May–October), a cool foggy season (November-January), and a dry season (February–April). According to XTBG internal ecological station meteorological data, the average annual precipitation at the experimental site is approximately 1560 mm, of which 85% takes place during the rainy season. The cool foggy season is misty at night and in the mornings. There is almost no mist or precipitation during the dry season, and the weather is dry and hot. The average annual temperature of the experimental site is 21.7 °C. The hottest month is July, with an average temperature of 25.5 °C, and the average temperature of the coldest month (January) is 14.8 °C.

In the wild orchid garden, native *Litsea liyuyingi*, *Litsea glutinosa*, *Lagerstroemia villosa*, *Mesua ferrea*, *Melia toosendan*, *Gardenia sootepensis*, and other woody dicotyledons are the main substrates on which *Dendrobium* and other epiphytic plants grow. To minimize confounding environmental effects, *Dendrobium* plants selected for this study had been growing in the wild orchid garden for several years and were fully adapted to their growth environment. Each species had at least 10 randomly located, independently growing and healthy plants in the garden. Based on the growth status of the plants and the availability of the plant materials, *Dendrobium polyanthum*, *D. cucullatum*, *D. loddigesii*, *D. crepidatum*, *D. chrysotoxum*, *D. fimbriatum*, *D. thyrsiflorum*, and *D. jenkinsii* were selected as the tested species. The eight species are the dominant *Dendrobium* plants growing in the orchid garden. They are ecologically adapted since they have better growth performances in the orchid garden than other *Dendrobium* species. The target species belong to two classifications (sect. *Chrysotoxae* and sect. *Dendrobium*) with different appearances. In addition, based on the phylogram of 19 *Dendrobium* species presented in our former paper, the eight species used in the present study are distributed in different clades (Additional file 1: Figure S1). Therefore, the target species here can reflect the genus in terms of general ecological and genetic adaptive strategies to a certain extent.

### Determination of traits

All detected traits were listed in additional file 2: Table S1. For each species, six individual plants with good growth conditions were selected. During the flowering period of *Dendrobium* from March to April, flower number per pseudobulb (FN) was measured directly in situ. Then, three flowers were selected from each plant (18 flowers per species), and the flowers along with their pedicels were taken and placed inside a self-sealing bag containing moistened paper balls. The flowers were quickly placed in a cooler and brought to the nearest laboratory for weighing to obtain flower fresh mass (FFM) and flower petal vein number (FPVN). Then, the flowers were placed in a cowhide envelope and placed in an oven at 70 °C for 48 h until they reached a constant mass to obtain flower dry mass (FDM). Flower water content (FWC) was calculated as (FFM–FDM)/FFM × 100%.

During the growth period of *Dendrobium* in August, the length of five pseudobulbs (PL) of each plant (30 values per species) were measured in situ with a ruler, and the five-pseudobulb width (PW) and five-pseudobulb internode length (PIL) values of each plant (30 values per species) were measured in situ using a Vernier caliper (precision: 0.01 mm; Guanglu, Guilin, China).

Five healthy leaves were taken from each plant (30 leaves per species). The leaves were separated by plant specimen, placed in a self-sealing bag containing moistened paper balls, and then quickly placed in a cooler and brought to the nearest laboratory to measure the leaf traits. Leaf fresh mass (LFM) was directly determined with an electronic balance (one-thousandth level), and then two of the five leaves were placed in purified water for 48 h to obtain leaf saturated mass (LSM). The leaf areas (LAs) of the other three leaves were measured by a Li-Cor 3000A area meter (Li-Cor Inc., Lincoln, NE, USA); then, the leaves were placed in a cowhide envelope and dried in a 70 °C oven for 48 h until they reached a constant weight, which was leaf dry mass (LDM). Finally, the two leaves measured for LSM were also dried, and LDM was measured. Leaf water content (LWC) was calculated as (LFM–LDM)/LFM × 100%; leaf dry matter content (LDMC) was calculated as LDM/LSM × 100%; and and specific leaf area (SLA) was calculated as LDM/LA (leaf area per dry mass).

To characterize leaf anatomy, transverse sections at the midpoint of the leaves were hand-cut, stained for 1 min with 0.1% toluidine blue, rinsed with distilled water, and photographed under a DM2500 light microscope (Leica Inc., Bensheim, Germany). Mesophyll thickness (MT), upper epidermal cell size (UECS) and stomatal pore depth (SPD) were then measured from the digital photographs with ImageJ v.1.48 software (http://rsbweb.nih.gov/ij/).

The abaxial midpoints of the leaves were pasted onto pellucid enamels and then transferred to glass slides after drying. The stomatal prints on the enamels were photographed under a DM2500 light microscope, and stomatal traits were measured with ImageJ software. Stomatal density (SD) was measured as the number of stomata per unit area and was calculated as the mean value of 30 digital images for each species (5 images per plant). Stomatal length (SL) and stomatal width (SW) were averaged from 30 randomly selected stomata for each species. The stomatal area index (SAI) was estimated by the formula SD × SL^2^ (Sack et al. 2003). The stomatal volume (SV) was estimated by SL × SW × SPD. After measuring the stomata, we completely removed the enamel and slowly scraped off the mesophyll with a double blade, mounted it on slides, and photographed it. Total vein length was measured manually with ImageJ software, and vein density (VD) was calculated as total vein length per area.

### Building a phylogenetic tree

**Fig. 1:**
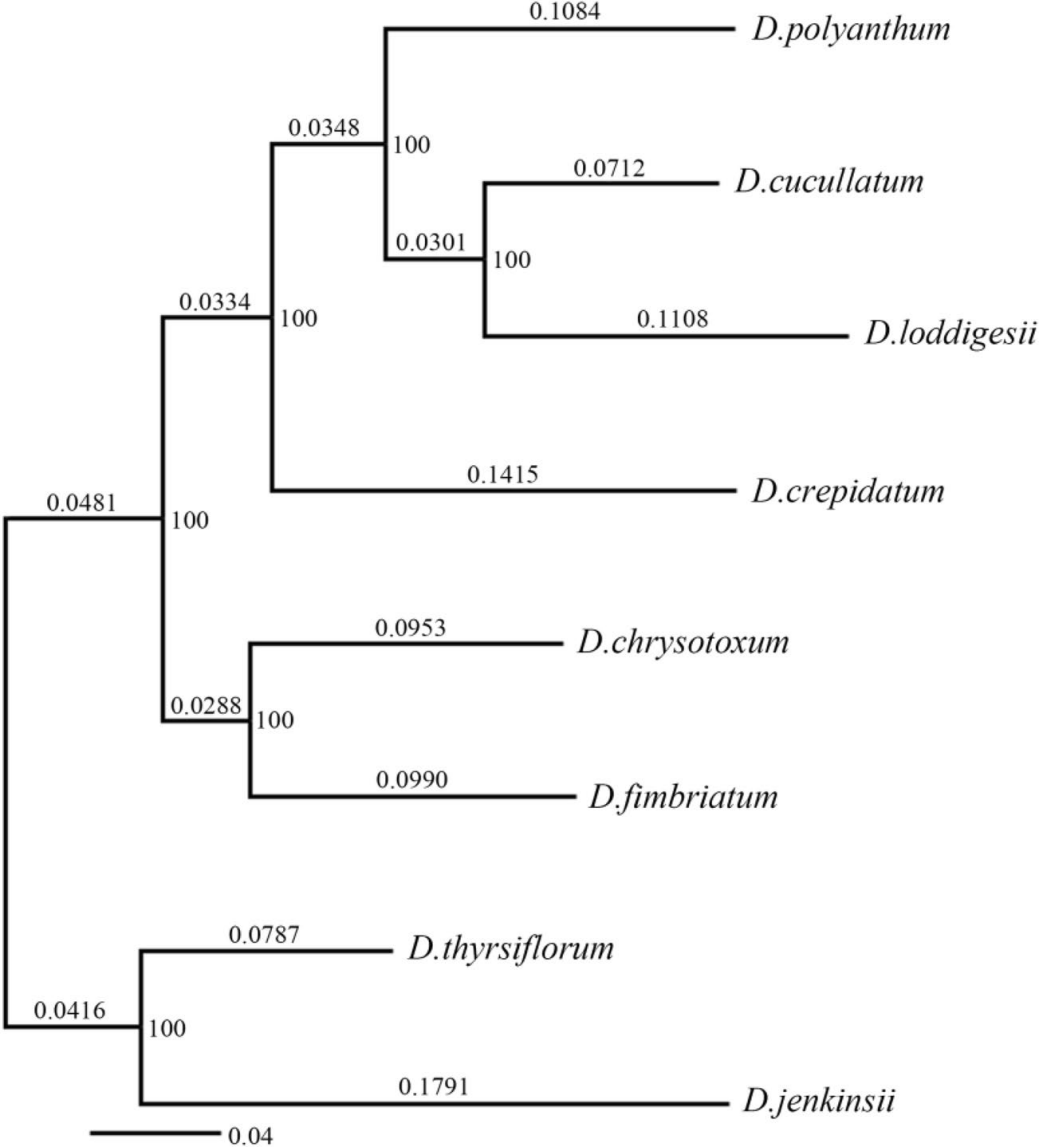
Phylogenetic tree of the eight *Dendrobium* species. The numerical values above the nodes on the phylogenetic tree indicate that the support rate for there being a split between species is greater than 50%, and the value on the phylogenetic tree branch represents the distance between two adjacent nodes

The phylogenetic tree in this study was constructed based on splicing nuclear ITS and *rbc*L sequences and chloroplast *matK* and *ycf5* sequences. These gene sequences were downloaded from GenBank (http://www.ncbi.nlm.nih.gov). Because the genus *Dendrobium* is closely related to *Bulbophyllum* (Freudenstein and Rasmussen 1999), this study selected *Bulbophyllum odoratissimum* as the outgroup. Sequence alignment was performed using the “CLUSTALW” module of MEGA v.5.0 software. Model selection was performed using ModelTest v.3.7 software, and the optimal model was selected using the Akaike information criterion. The GTR (general time reversible) + G (gamma shape) model was the optimal model for the dataset in this study. Using MrBayes v.3.2 software, phylogenetic analysis of the gene sequence matrix was carried out by the Bayesian method, and a phylogenetic tree was constructed. The analysis was run 100,000 times, and a relatively stable phylogenetic tree (Fig. 1) was selected for the study. A posttest was used to estimate the stability of the nodes.

### Statistical analysis

Prior to analysis, all plant trait data were log10 transformed to increase normality and variance homogeneity. The statistical analyses were mainly performed using R v.3.01 (http://ftp.ctex.org/mirrors/CRAN/). The “vegan” package in R software was used to perform principal component analysis (PCA) of the species mean values of all traits and explore the main relationships between plant trait changes on the first two major axes.

To assess the phylogenetic conservation of traits, we first applied the “picante” package in R software (Kembel et al. 2010) to detect the phylogenetic signal of these traits (*K* value) based on the *K*-statistics, which is based on the assumption of Brownian motion trait evolution. *K *> 1 indicates that the trait is more conserved and is strongly influenced by phylogeny; *K *< 1 indicates that the variability of the trait is higher and is less affected by phylogeny; and *K *= 1 indicates that the trait follows the random variation found in the Brownian motion model (Blomberg et al. 2003).

Phylogenetic independent contrast (PIC) analysis first uses the analysis of traits (AOT) module of Phylocom software to calculate the node contrast values of leaf traits (Webb et al. 2008). These contrasts were calculated as trait differences between two sister species pairs at the tips and were subsequently weighted to obtain an internal node average. Then, they were divided by the expected amount of change, which was calculated as the square root of the branch length separating the two taxa. These comparisons provide N − 1 (N refers to the number of species, N = 8 in this study) independent data points, with each point representing evolutionary divergence (Ackerly 1999; Ackerly and Reich 1999). Then, using Pearson correlation analysis in R software, we calculated the correlations between traits before and after PIC analysis.

## Results

### Species mean correlations between traits

**Fig. 2:**
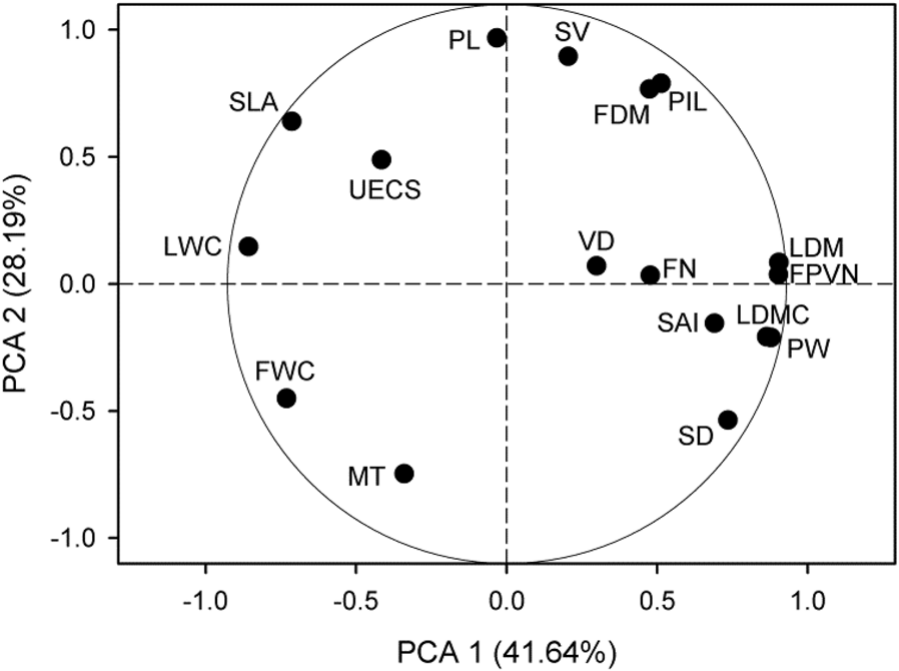
Principal component analysis (PCA) of species mean values of *Dendrobium* plant traits. *LDM* leaf dry mass, *LWC* leaf water content; *LDMC*, leaf dry matter content, *SLA* specific leaf area, *MT* mesophyll thickness, *UECS* upper epidermal cell size, *SD* stomatal density, *SV* stomatal volume, *SAI* stomatal area index, *VD* vein density, *PL* pseudobulb length, *PW* pseudobulb width, *PIL* pseudobulb internode length, *FDM* flower dry mass, *FWC* flower water content, *FPVN* flower petal vein number, *FN* flower number per pseudobulb

PCA of the species mean traits shows that the first two axes explain 41.64% and 28.19% of the total trait changes, respectively (Fig. 2). The first major axis is significantly positively correlated with leaf dry mass, leaf dry matter content, stomatal density, pseudobulb width and flower petal vein number and is significantly negatively correlated with leaf water content, specific leaf area and flower water content (Table 1). The second major axis shows a significant positive correlation with stomatal volume, pseudobulb length, pseudobulb internode length and flower dry mass and is significantly negatively correlated with mesophyll thickness (Table 1).

**Table 1:** Correlation coefficients (*r*) of the first two axes of PCA and phylogenetic signals (*K*) for the functional traits of *Dendrobium* species in this study

Functional traits	Codes	Units	Species mean value PCA		Phylogenetic signals	
			First major axis *r*	Second major axis *r*	*K*	*P*
Leaf dry mass	LDM	mg	0.904^**^	0.085	*1.163*	*0.044*
Leaf water content	LWC	%	− 0.857^**^	0.144	*1.531*	*0.007*
Leaf dry matter content	LDMC	%	0.863^**^	− 0.209	*1.720*	*0.009*
Specific leaf area	SLA	cm^2^/g	− 0.714^*^	0.640	*1.415*	*0.019*
Mesophyll thickness	MT	μm	− 0.341	− 0.747^*^	0.931	0.138
Upper epidermal cell size	UECS	μm^2^	− 0.415	0.488	0.941	0.363
Stomatal density	SD	no./mm^2^	0.735^*^	− 0.535	*1.537*	*0.005*
Stomatal volume	SV	μm^3^	0.204	0.895^**^	0.984	0.096
Stomatal area index	SAI	10^−2^ no.	0.689	− 0.155	*1.108*	*0.046*
Vein density	VD	mm/mm^2^	0.301	0.071	0.862	0.366
Pseudobulb length	PL	cm	− 0.032	0.967^***^	*1.022*	*0.041*
Pseudobulb width	PW	mm	0.878^**^	− 0.211	*1.026*	*0.042*
Pseudobulb internode length	PIL	10^−1^ mm	0.513	0.789^*^	0.904	0.241
Flower dry mass	FDM	mg	0.474	0.767^*^	*1.143*	*0.025*
Flower water content	FWC	%	− 0.748^*^	− 0.457	0.888	0.204
Flower petal vein number	FPVN	no.	0.903^**^	0.037	0.921	0.393
Flower number	FN	no.	0.477	0.033	0.906	0.607

*K *≥ 1 refers to changes in traits that are strongly controlled by phylogeny

*K *< 1 reflects traits that are less affected by phylogeny

*p < 0.05

**p < 0.01

### Phylogenetic conservation of traits

The water-related traits of the *Dendrobium* plants in this study vary significantly along the phylogenetic tree (Fig. 1), suggesting that phylogeny affects the changes in the water-related traits of *Dendrobium*. Of these, leaf dry mass, leaf water content, leaf dry matter content, specific leaf area, stomatal density, stomatal area index, pseudobulb length, pseudobulb width, and flower dry mass showed phylogenetic signals (*K* values > 1), indicating that these traits are strongly influenced by phylogeny, while the other studied traits had *K* values < 1, indicating that the phylogenetic signals are weaker, as these traits are more affected by environmental factors (Table 1).

### Evolutionary associations between traits

**Fig. 3:**
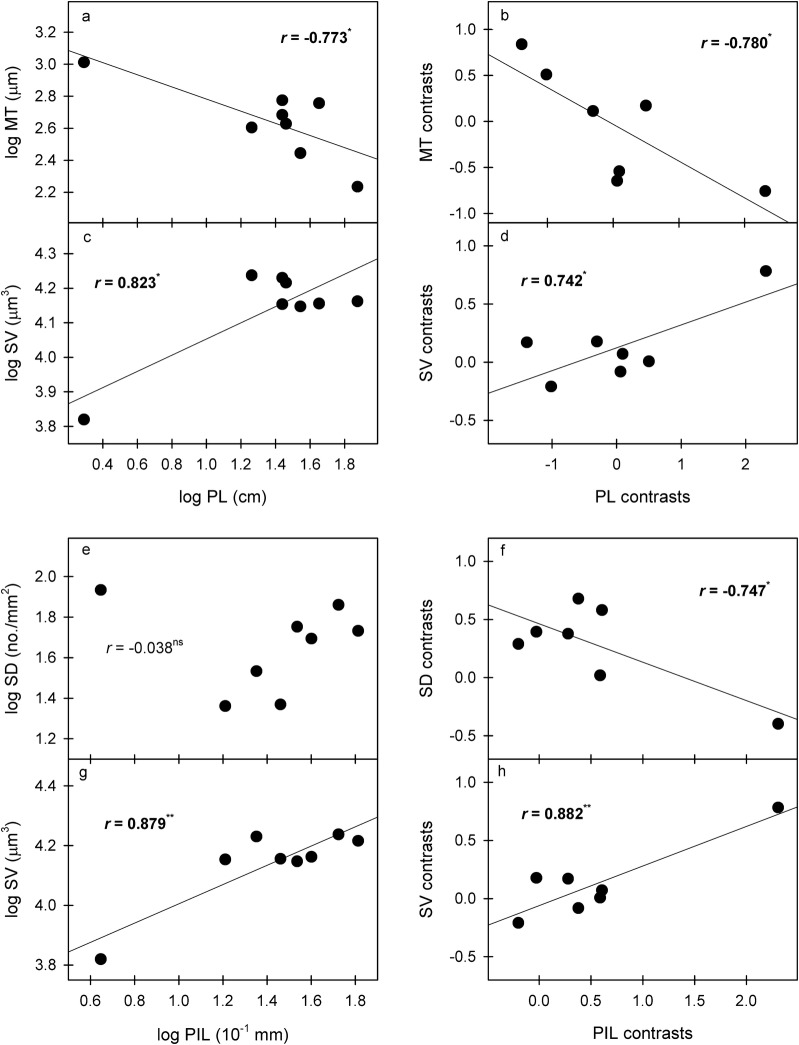
Significant correlations of species mean values and phylogenetically independent contrasts (PICs) between pseudobulb and its internode length and leaf traits. *p < 0.05; **p < 0.01. *MT* mesophyll thickness, *SD* stomatal density, *SV* stomatal volume, *PL* pseudobulb length, *PIL* pseudobulb internode length

**Fig. 4:**
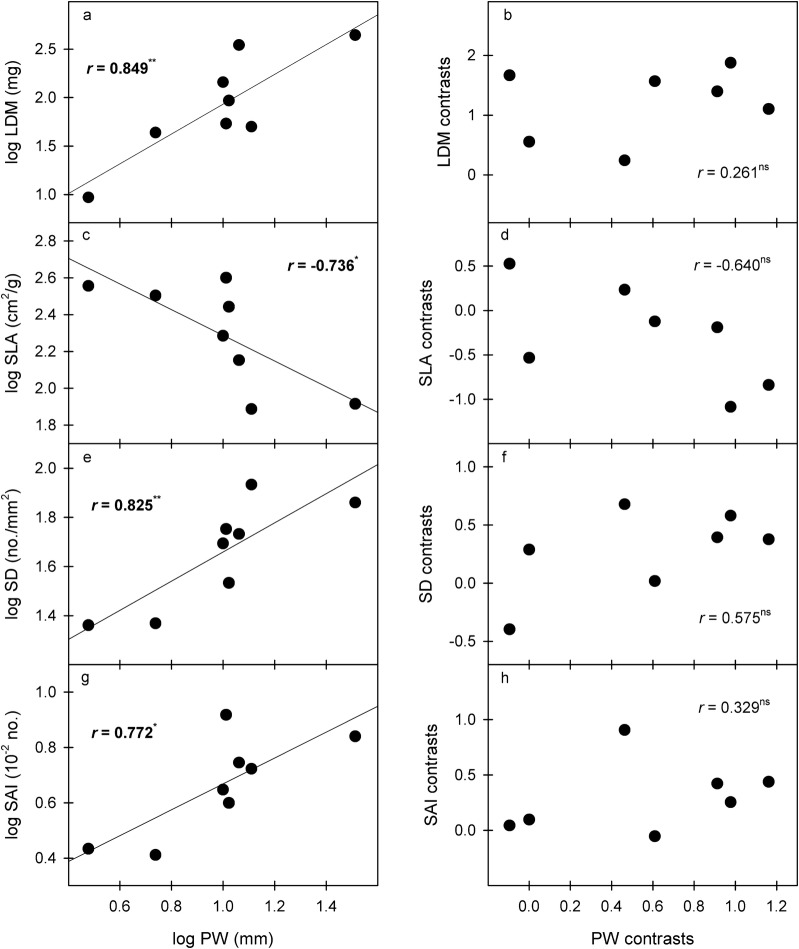
Significant correlations of species mean values and PICs between pseudobulb width (PW) and leaf traits. *p < 0.05; **p < 0.01. *LDM* leaf dry mass, *SLA* specific leaf area, *SD* stomatal density, *SAI* stomatal area index

**Fig. 5:**
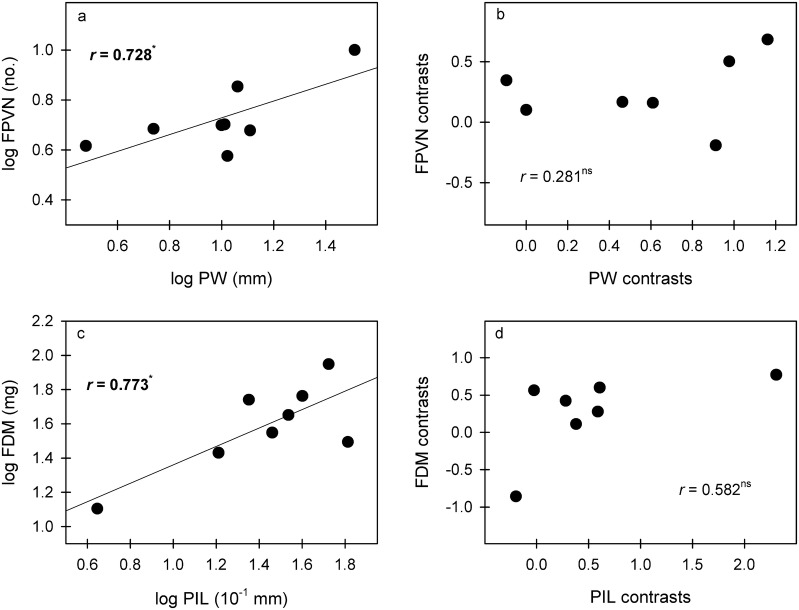
Significant correlations of species mean values and PICs between pseudobulb traits and flower traits. *p < 0.05; ** *p *< 0.01. *PW* pseudobulb width, *PIL* pseudobulb internode length, *FDM* flower dry mass, *FPVN* flower petal vein number

Pseudobulb length was positively correlated with SV and PIL but was negatively correlated with MT; PIL was also positively correlated with SV (Fig. 3; Table 2). After phylogeny was considered, these correlations were still significant (Fig. 3). Phenotypically, PW was significantly positively correlated with LDM, SD and SAI but was negatively correlated with SLA. After the phylogenetic effects were eliminated, the correlations between PW and leaf traits were all nonsignificant (Figs. 4 and 5). In contrast, the correlation between PIL and SD was nonsignificant for species values but significant and negative after correcting for phylogeny (Fig. 3).

**Table 2:** Pairwise cross-species and phylogenetically independent contrast (PIC) correlations between the traits in this study

	LDM	LWC	LDMC	SLA	MT	UECS	SD	SV	SAI	VD	PL	PW	PIL	FDM	FWC	FPVN	FN
LDM		− 0.336	0.209	− 0.176	− 0.190	− 0.187	− 0.442	0.561	− 0.610	0.229	0.459	0.261	0.480	0.703	− 0.387	0.222	0.322
LWC	− 0.689		- 0.986^***^	0.501	0.476	− 0.286	− 0.427	0.178	− 0.032	− 0.806^*^	− 0.062	− 0.235	− 0.007	− 0.156	0.420	− 0.287	− 0.003
LDMC	0.706^*^	− 0.987^***^		− 0.445	− 0.503	0.307	0.435	− 0.240	0.054	0.742^*^	0.063	0.108	− 0.008	0.025	− 0.347	0.250	0.029
SLA	− 0.594	0.743^*^	− 0.757^*^		− 0.505	0.435	− 0.534	0.469	0.065	− 0.547	0.704	− 0.640	0.556	0.153	0.314	− 0.434	− 0.046
MT	− 0.277	0.400	− 0.363	− 0.298		− 0.754^*^	0.107	− 0.408	− 0.173	− 0.292	− 0.780^*^	0.338	− 0.628	− 0.432	0.238	0.063	0.060
UECS	− 0.513	0.210	− 0.272	0.668	− 0.575		0.368	0.054	0.657	0.387	0.327	− 0.033	0.094	0.288	0.035	− 0.411	− 0.673
SD	0.595	− 0.723^*^	0.783^*^	− 0.776^*^	− 0.006	− 0.323		− 0.726^*^	0.729^*^	0.702	− 0.693	0.575	− 0.747^*^	− 0.225	− 0.048	− 0.013	− 0.686
SV	0.354	0.096	− 0.145	0.395	− 0.545	0.130	− 0.403		− 0.272	− 0.256	0.742^*^	− 0.224	0.882^**^	0.726^*^	− 0.448	0.226	0.391
SAI	0.587	− 0.503	0.575	− 0.414	− 0.262	− 0.100	0.857^**^	− 0.042		0.424	− 0.323	0.329	− 0.325	0.042	− 0.151	− 0.033	− 0.739^*^
VD	0.008	− 0.542	0.428	− 0.252	− 0.391	0.444	0.244	− 0.122	0.104		− 0.189	0.696	− 0.204	0.375	− 0.529	0.329	− 0.406
PL	0.092	0.103	− 0.155	0.647	− 0.773^*^	0.461	− 0.550	0.823^*^	− 0.221	0.007		− 0.525	0.906^**^	0.518	− 0.135	− 0.059	0.324
PW	0.849^**^	− 0.670	0.680	− 0.736^*^	− 0.063	− 0.435	0.825^**^	0.037	0.772^*^	0.244	− 0.280		− 0.439	0.413	− 0.395	0.281	− 0.478
PIL	0.608	− 0.240	0.232	0.167	− 0.693	− 0.028	− 0.083	0.879^**^	0.235	− 0.097	0.777^*^	0.253		0.582	− 0.446	0.311	0.546
FDM	0.526	− 0.227	0.143	0.128	− 0.640	0.229	− 0.042	0.823^**^	0.239	0.333	0.666	0.418	0.773^*^		− 0.611	0.267	− 0.097
FWC	− 0.570	0.547	− 0.485	0.295	0.506	0.070	− 0.234	− 0.570	− 0.362	− 0.480	− 0.331	− 0.534	− 0.675	−0.749^*^		− 0.883^**^	− 0.282
FPVN	0.765^*^	− 0.656	0.671	− 0.626	− 0.196	− 0.546	0.547	0.273	0.555	0.160	− 0.031	0.728^*^	0.553	0.408	−0.821^**^		0.534
FN	0.535	− 0.323	0.368	− 0.389	0.038	− 0.756^*^	0.053	0.283	− 0.032	− 0.383	0.097	0.186	0.499	0.017	−0.355	0.653	

Correlation data are given for species-based analyses below the diagonal and for PIC analyses above the diagonal. The degree of significance for each correlation is indicated as follows

*LDM* leaf dry mass, *LWC* leaf water content; *LDMC*, leaf dry matter content, *SLA* specific leaf area, *MT* mesophyll thickness, *UECS* upper epidermal cell size, *SD* stomatal density, *SV* stomatal volume, *SAI* stomatal area index, *VD* vein density, *PL* pseudobulb length, *PW* pseudobulb width, *PIL* pseudobulb internode length, *FDM* flower dry mass, *FWC* flower water content, *FPVN* flower petal vein number, *FN* flower number per pseudobulb

^*^p < 0.05

^**^p < 0.01

^***^p < 0.001

Phenotypically, PW was significantly positively correlated with FPVN, and PIL was positively correlated with FDM, while these correlations were insignificant after correcting for phylogeny (Figs. 3 and 5).

**Fig. 6:**
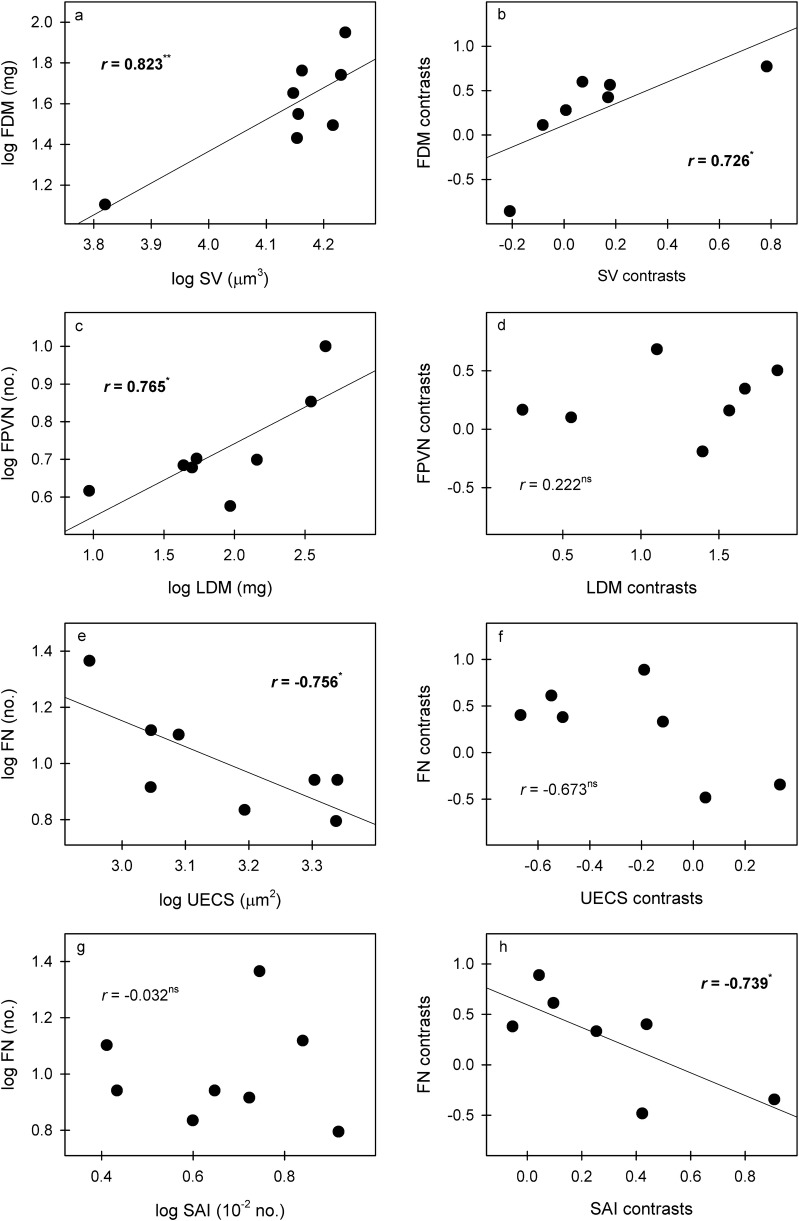
Significant correlations of species mean values and PICs between leaf traits and flower traits. *p < 0.05; **p < 0.01. *LDM* leaf dry mass, *UECS* upper epidermal cell size, *SV* stomatal volume, *SAI* stomatal area index, *FDM* flower dry mass, *FPVN* flower petal vein num ber, *FN* flower number per pseudobulb

Phenotypically and phylogenetically, FDM was positively correlated with SV (Fig. 6a–b), while FWC was negatively correlated with FPVN (Table 2). FPVN was phenotypically but not phylogenetically positively correlated with LDM (Fig. 6c–d). FN was phenotypically negatively correlated with UECS and phylogenetically negatively correlated with SAI (Fig. 6e–h).

Considering leaf traits, LDMC was positively correlated with LDM but was negatively correlated with LWC; meanwhile, SLA was positively correlated with LWC but was negatively correlated with LDMC (Table 2). After the phylogenetic effects were eliminated, LDMC and LWC were still significantly correlated. SD was significantly correlated with LWC, LDMC, and SLA, but these correlations were all nonsignificant after correcting for phylogeny (Table 2). Phenotypically and phylogenetically, SD was positively correlated with SAI. In addition, SD was phylogenetically negatively correlated with SV, UECS was phylogenetically negatively correlated with MT, and VD was positively correlated with LDMC but negatively correlated with LWC after phylogeny was considered (Table 2).

## Discussion

Environmental and phylogenetic factors affect the water-related traits of various structures in plants from the genus *Dendrobium* (Table 1). This is consistent with our first hypothesis and our previous findings on the morphological and structural traits of *Dendrobium* leaves (Sun et al. 2014) and nutrient traits (Sun et al. 2019), indicating that the functional traits of various structures of *Dendrobium* are affected by phylogeny and the environment. When studying the functional adaptability of this genus, phylogenetic factors should be considered (Sun et al. 2019). LDM, LWC, LDMC, SLA, SD, SAI, PL, PW and FDM in the *Dendrobium* genus are strongly influenced by phylogeny (Table 1), indicating that changes in these traits over the course of the evolution of this genus were more conservative than the hypothetical random variation model and that their changes are mainly influenced by phylogeny (Blomberg et al. 2003; Hodges and Derieg 2009; Sun et al. 2019). In contrast, the phylogenetic signals of MT, UECS, SV, VD, PIL, FWC, FPVN and FN are weak (*K *< 1) (Table 1), indicating that these traits are mainly influenced by environmental factors (Dunbar-Co et al. 2009; Zhang et al. 2015). Studies on *Paphiopedilum*, another orchid genus, reached slightly different consistent conclusions, but some of the findings of these studies are consistent with our results, such as the phylogenetic signals of mesophyll thickness, upper epidermal cell size and vein density being weak; however, some other findings of these studies were contrary to our results, such as the phylogenetic signals of stomatal size and flower number being strong but the phylogenetic signals of specific leaf area, stomatal density and the stomatal area index being weak (Zhang et al. 2012, 2017). The discrepancy between our observations and those in *Paphiopedilum* are probably related to the habits of the plant materials tested. *Paphiopedilum* are terrestrial and C3 plants, while the *Dendrobium* plants studied here are all epiphytic and facultative CAM plants that have lower dry matter accumulation rates than C3 plants. *Dendrobium* plants have concomitant C3 and CAM photosynthesis patterns (He et al. 1998; Zhang et al. 2014). Drought stress induces the CAM pattern, and concomitant C3 and CAM patterns are found when plants are rewatered (He et al. 1998). Drought stress can play a key role in shaping diversity, such as increasing taxonomic diversity (Araújo and Santos 2019). *Dendrobium*, one of the most diverse epiphytic genera of Orchidaceae, has high drought tolerance (Stern et al. 1994; Ji 1999). Family Orchidaceae is relatively old, and its fossil record can be traced back to the early Miocene (Conran et al. 2009). Our research and previous studies on other orchid species suggest that although orchids have naturally become differentiated during their long evolutionary process, morphological changes in orchids have been influenced by phylogeny.

The water-related traits and their relationships that mediate the response of a species to environmental changes have helped clarify the adaption of plants to environmental stress (Dunbar-Co et al. 2009; Sun et al. 2017). Most studied traits, including LDM, LWC, LDMC, SLA, MT, SD, SV, PL, PW, PIL, FDM, FWC, and FPVN, were mainly distributed along the first two principal component axes (Fig. 2; Table 1). When combining the above PCA-significant traits with the traits that have strong phylogenetic signals, we find that leaf traits including leaf dry mass, leaf water content, leaf dry matter content, specific leaf area and stomatal density; pseudobulb length and width; flower dry mass and the functional coordination between the traits are the main ways by which these plants have adapted to a water-deficient environment.

We hypothesized that water-related stress would act as an environmental filter, resulting in some trait relationships among the pseudobulbs, leaves and flowers of *Dendrobium* species. The trait correlations between pseudobulbs and leaves indicate their functional coordination and reflect different strategies for plant adaptation to drought (Yang et al. 2016; Li and Zhang 2019). In *Dendrobium*, the larger the pseudobulb is, the more water storage tissue the pseudobulb contains, the larger the water storage cells, and the greater the water storage capacity (Li and Zhang 2019). Therefore, plants with larger pseudobulbs (higher PL, PW and PIL values) with lower mesophyll thickness and stomatal density and corresponding higher stomatal volume (Figs. 3 and 4, Table 2) exemplify that these plants mainly adapt to drought through the pseudobulb reservoir strategy (Yang et al. 2016). In contrast, plants with shorter and finer pseudobulbs with higher mesophyll thickness and stomatal density and lower stomatal volume adapt to drought through water retention in the leaves (mainly cuticle and mesophyll water retention and stomatal regulation) (Yang et al. 2016). For example, the pseudobulb of *D. jenkinsii* in this study is the shortest and has the lowest stomatal volume and highest mesophyll thickness and stomatal density, reflecting the typical leaf water retention strategy for water-deficient environments. In fact, a long pseudobulb tends to increase the water transportation distance and thus should increase the conduction resistance of water and nutrients in the pseudobulb, thus resulting in lower water conductance and transport rates (Sack and Frole 2006; Sack and Holbrook 2006). In contrast, short and wide vessels tend to have higher water conductance and transport rates but are also more vulnerable to cavitation and deformation (Sack and Frole 2006; Sack and Holbrook 2006). Here, we suspect that a trade-off exists between increased water conductance and decreased vessel vulnerability in *Dendrobium* pseudobulbs. This should be another reason for the stronger reservoir water storage in larger pseudobulbs. Under the same conditions that lead to leaf loss, water loss from the pseudobulb is much lower than that under conditions where the leaves remain intact (Zotz and Tyree 1996; Li and Zhang 2019). Higher stomatal density and correspondingly lower stomatal size (SV here) usually support more and faster leaf gas exchange and leaf water transpiration (Franks and Beerling 2009). Therefore, plants that adapt to drought mainly through the pseudobulb reservoir strategy should be more efficient than those that adapt to drought mainly through the leaf water retention strategy.

Positive correlations of species values exist between pseudobulb width and flower petal vein number and between pseudobulb internode length and flower dry mass, indicating that these correlations also have important ecological significance during the process of adapting to environmental changes (Fig. 5). *Dendrobium* flowers grow directly on the surface of the pseudobulb, and the large amounts of water, nutrients and hormones that are required for flower formation, opening and display may be obtained directly from pseudobulbs. In addition, the main function of *Dendrobium* flowers, attracting insect pollination and reproduction, requires a large amount of water. The plants must also be maintained in an erect state, and the larger the pseudobulb, the more fully the flower is displayed. Pseudobulbs play a role in water, nutrient and hormone storage, supporting other structures (leaves, flowers, and roots), and maintaining the plant’s erect morphology (Juenger et al. 2005). Therefore, larger pseudobulbs are key to ensuring a full flower display and attracting insects for pollination.

There are certain significant relationships between leaf and flower traits, indicating similar selective pressures between the leaves and flowers of *Dendrobium*. Stomatal volume is positively correlated with flower dry mass while the stomatal area index is negatively correlated with flower number per pseudobulb according to PIC values (Fig. 6). As we explained above, plants with more (larger stomatal density and stomatal area index) and smaller (lower stomatal volume) stomata in *Dendrobium* mainly adapt to drought through water retention in the leaves; thus, far fewer flowers and much lower flower dry mass should be associated with the leaf water retention strategy. High stomatal density, a large upper epidermal cell size, and small stomatal volume all tend to support high and fast leaf gas exchange and water transpiration (Franks and Beerling 2009; Zhang et al. 2015). Large amounts of water loss through leaves may induce further physiological drought and thus limit plant growth, including the growth of flowers (low flower dry mass and a small flower number per pseudobulb) and pseudobulbs (low pseudobulb length and width) (Additional file 2: Table S1)

Significant correlations also exist between different water-related traits in a structure. Some of these correlations may be because of autocorrelation, such as LDMC being positively correlated with LDM but negatively correlated with LWC, SLA being positively correlated with LWC but negatively correlated with LDM, SD being positively correlated with SAI, PL being positively correlated with PIL, and FDM being negatively correlated with FWC (Table 2). Some common conclusions usually reached for woody plant groups are also confirmed here. For example, species values of stomatal density and vein density are positively correlated with LDMC but negatively correlated with LWC and SLA (Table 2), reflecting leaf hydraulic balance, since dense leaves increase resistance to the diffusion of water and CO_2_, which can be ameliorated by increasing stomatal density and vein density to maintain a high photosynthetic rate (Sack and Frole 2006; Poorter et al. 2009; Sun et al. 2014). Similar to the correlation between leaf vein density and leaf water content, flower petal vein number is also negatively correlated with flower water content (Table 2), reflecting that a similar water balance should also exist in flowers. The low flower water content may also be ameliorated by increasing flower petal vein number to maintain a long flower display. These two correlations exist in the evolutionary history of *Dendrobium*, with important ecological functions in maintaining photosynthetic production and attracting insect pollinators (Ackerly and Reich 1999; Ackerly et al. 2000).

## Conclusions

Environmental and phylogenetic factors affect the water-related traits of various structures in *Dendrobium* plants. Leaf dry mass, leaf water content, leaf dry matter content, specific leaf area, stomatal density, stomatal area index, pseudobulb length, pseudobulb width, and flower dry mass of *Dendrobium* are strongly influenced by phylogeny. Significant correlations between the pseudobulbs, leaves and flowers of *Dendrobium* species indicate their functional coordination and reflect different strategies for plant adaptation to drought. A trade-off exists in plants between two drought tolerance strategies: pseudobulb storage and leaf water retention. Plants with larger pseudobulbs, thinner blades, lower stomatal density, and larger stomata mainly adapt to drought through the pseudobulb reservoir strategy. In contrast, plants with shorter and finer pseudobulbs, thicker blades, higher stomatal density, and smaller stomata adapt to drought through water retention in the leaves. The production of many small flowers and lower flower dry mass should be associated with the leaf water retention strategy. In addition, an obvious water balance exists in both the leaves and flowers of *Dendrobium* plants. Future research should carry out more experiments in other epiphytic groups to further detect the drought endurance relationships between different structures and to discover the water balance mechanisms in flowers.

## Supplementary information

**Additional file 1: Figure S1.** The phylogram of 19 *Dendrobium* species. The phylogenetic tree was cited from Sun et al. 2014. Target species in this study were marked by boxes.

**Additional file 2: Table S1.** The studied plant species and their water-related traits.

## Data Availability

The data that support the findings of this study are available from the corresponding author upon reasonable request.

## Abbreviations

LDM: Leaf dry mass
LWC: Leaf water content
LDMC: Leaf dry matter content
SLA: Specific leaf area
MT: Mesophyll thickness
UECS: Upper epidermal cell size
SD: Stomatal density
SV: Stomatal volume
SAI: Stomatal area index
VD: Vein density
PL: Pseudobulb length
PW: Pseudobulb width
PIL: Pseudobulb internode length
FDM: Flower dry mass
FWC: Flower water content
FPVN: Flower petal vein number
FN: Flower number per pseudobulb
