# Supplementary material for: Evolutionary correlation of water-related traits between different structures of Dendrobium plants
Source: Bot Stud. 2020 May 16;61:16. doi: 10.1186/s40529-020-00292-4 (PMC7230118; doi:10.1186/s40529-020-00292-4)
Supplement: Supplementary file 1 — Additional file 1: Figure S1. The phylogram of 19 Dendrobium species. The phylogenetic tree was cited from Sun et al. 2014. Target species in this study were marked by boxes. [file 40529_2020_292_MOESM1_ESM.docx]

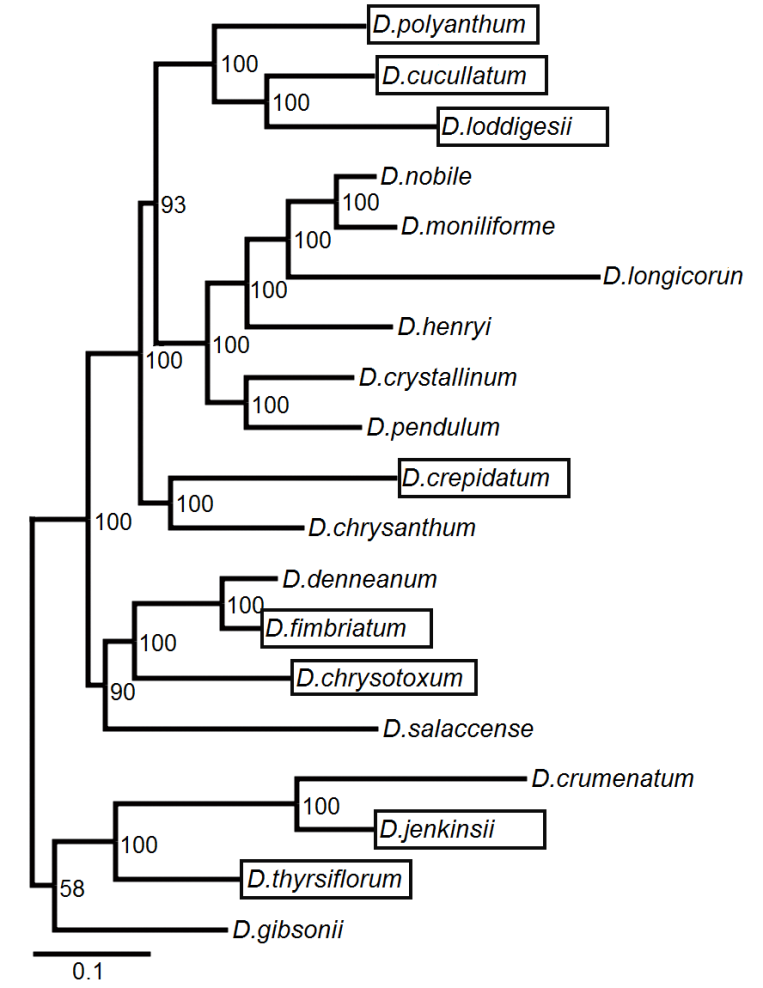


**Fig. S1** The phylogram of 19 *Dendrobium* species. The phylogenetic tree was cited from Sun et al. 2014. Target species in this study were marked by boxes.
